# Adrenalectomy by laparoscopic anterolateral transperitoneal approach for patients with previous abdominal surgery

**Published:** 2012

**Authors:** R Ganescu, S Paun, M Beuran, M Vartic, D Paun, C Dumitrache

**Affiliations:** * Emergency Hospital Bucharest, Romania; ** “C.I. Parhon” National Institute of Endocrinology, Bucharest, Romania

**Keywords:** Laparoscopic adrenalectomy, previous abdominal surgery, adrenal pathology, transperitoneal approach

## Abstract

Adrenal surgery has been radically changed by laparoscopic approach and we wonder whether the increase in the number of adrenalectomies is entirely justified by better understanding of the pathology and a developed diagnosis methods. The type of approach (transabdominal/retroperitoneal) remains a matter of the surgeon’s experience.

**Method:** In the past 8 years, we have performed more than 200 laparoscopic adrenalectomies by transperitoneal approach, 24 of them having previously significant abdominal surgery (cholecistectomy, gastric surgery, colectomy, bowel obstruction, exploratory laparoscopy, and adrenalectomy). The patients had a variety of adrenal pathologies such as Cushing disease, Cushing syndrome, Conn syndrome, incidentaloma, pheochromocytoma and even carcinoma.

**Results:** 3 cases were converted to open approach, only one because of the adhesions. Reasons for conversion were also: spleen intarctisation and a difficulty in mobilizing the tumor. Operating time was not significantly prolonged because of the adhesions (40-360 min, median time 127 min). Postoperative evolution was simple with no morbidity or mortality and a fast recovery was recorded.

**Conclusions:** Choosing the type of approach is related to surgeon experience, although 79-94% of the surgeons prefer the transabdominal lateral approach. We believe that with an experienced surgical team, there is no difficulty in performing adrenalectomy by transabdominal approach, with no significantly prolonged operating time, even though the patient has previously had abdominal surgery.

## Introduction

Since M. Gagnier [**1**] first performed the laparoscopic adrenalectomy, in 1992, up to the present, the indications for this type of surgery were modified and enlarged and one of the debates was about laparoscopic approach for patients with previous abdominal surgery. In past decade, laparoscopic adrenalectomy has become the “gold standard” for adrenal pathology, regardless of the catecholamine level for tumors smaller then 6 cm, although we believe that even a larger tumor (functioning or nonfunctioning tumor) can be safely removed by laparoscopic approach if there is no contraindication for laparoscopy [**2**].

Previous abdominal surgery remains a challenge for the surgeon when there is a need to perform a laparoscopic adrenalectomy, and also for an open approach, because of the adhesions. The most important problems in laparoscopic adrenalectomy for patients with previous abdominal surgery is creating pneumoperitoneu and working space and then, multiple adhesions that can prolong the operating time, increase technical difficulty and, also, a higher risk of iatrogenic lesions appears [**3**]. There is also a matter of the surgeon’s experience regarding the type of approach that is used. Most surgeons are more comfortable with transabdominal approach at least for the reason that they are very familiar with the abdominal cavity and in case of conversion to open surgery they can perform the surgery faster and safer for patient. Adrenal tumors are rare and adrenalectomy is an uncommon surgery even if it is performed by open or laparoscopic approach, therefore, there are only a few centers that can perform this type of surgery with best results.

Although there are surgeons who approach adrenal gland retroperitoneal in patients with previous abdominal surgery, there is no specific contraindication for the transabdominal approach (some authors consider it a “relative contraindication”) [**4-6**].

Adrenal tumor size and local invasions are also a relative contraindication if we think about the laparoscopic adrenalectomy safely performed for tumors larger then 10 cm and also for carcinoma with no morbidity or mortality and a fast recovery [**1**]. The advantages of the laparoscopic approach can also be consistent in adrenal pathology. Minimally intraoperative blood loses, low postoperative pain, cosmetic appearance, fast recovery, perioperative complications are only the most significant advantages.

Because, in general, this is the type of surgery for adults, there are many patients with a previous abdominal surgery (open or laparoscopic approach). In our study, we considered only the patients with a previous surgery in the upper abdomen (not including appendectomy or gynecologic surgery) and we tried to demonstrate that there is no significant difference regarding the operating time, conversion rate or postoperative recovery and hospital stay.

## Material and method

We retrospectively reviewed 8 years of experience (2003-2011) in laparoscopic adrenalectomy in a single surgical department in Emergency Hospital Bucharest. In this period of time, the same surgical anesthesia team performed more than 200 laparoscopic adrenalectomys only by anterolateral transperitoneal approach, 23 of them for patients (one of them had 2 consecutive operations for left and right side) with previously abdominal surgery in upper abdomen. Our surgical-anesthesia team is trained in advanced laparoscopic surgery and has been performing laparoscopic adrenalectomy since April 2003, meaning that this study also includes the learning curve for this type of surgery.

The medical records for all 23 patients with 24 surgical procedures in this study were reviewed with concern to demographic, surgical procedure (including previous abdominal surgery), pathology and outcome. All the patients treated in our clinic were diagnosed and prepared for surgery by an endocrinologist from “C. I. Parhon” National Institute of Endocrinology, including the preoperative medication for the patient with pheocromocytoma and also postoperative survey.

## Results

From a total of 212 adrenalectomies, 200 of them were performed by laparoscopic approach in the Department of General Surgery of the Emergency Hospital Bucharest by the same surgical team, 24 patients have previously had an abdominal surgery in the upper abdomen with a demografic showed in **Table I**.

**Table I.** Demographic information

***Demographic***	***Women***	***Men***
**Nr.**	21	3
**Age**	49,71(27-72)	53,66 (47-61)
**Side: Left**	9	1
**Right**	11	2
**Bilateral**	1	0

Preoperative diagnosis was as in **Table II**.

**Table II.** Pathology

	***Women***	***Men***
**Cushing disease**	4(5 procedures)	1
**Cushing syndrome**	4	1
**Conn syndrome**	1	0
**Incidentaloma**	8	1
**Pheochromocytoma**	2	0
**Neoplasia**	1	0

The surgical procedure used for all the patients, once the patients’ informed consents have been granted, is supposed to be the transperitoneal anterolateral subcostal laparoscopic approach with 4 trocars of 10 mm, pneumoperitoneum of 12 mmHg with the operation table adjusted to expose the subcostal region and optical of 30°. During the first year of experience (5 cases in the studied group), the adrenal gland dissection was carried out with the monopole dissector - Hook and the adrenal central vena being clipped and then sectioned, subsequently (19 cases from the studied group); the dissection was carried out with LigaSure Altas sealing and sectioning the central vena with the aid of this tool.

These patients have previously had an abdominal surgery in the upper abdomen as showed in **Table III**.

**Table III.** Previously abdominal surgery

**Previously abdominal surgery**	**Number**
Cholecystectomy laparoscopic/open	4/3
Biliary surgery (open)	1
Hiatal hernia (open)	1(2 procedures)
Adrenalectomy (open)	3-relapse
Umbilical hernia, POVH (open)	2
Gastric surgery (open)	2
Colectomy (open)	1
Splenectomy (open)	1
Hepatic hydatic cyst (open)	1
Laparoscopy	1
Mesenteric infarctisation (open)	1
POVH (open)	2

The operating time was between 40 and 360 min (**Table IV**), median 127 min with no lengthening because of the postoperative adhesions (our median operating time in all 200 laparoscopic adrenalectomy was of 85 min [**7**]), but we encountered 3 conversions to open surgery because of: adhesion after hiatal hernia, spleen infartisation who needed also a splenectomy and inflammatory adhesions with a very difficult mobilization of the specimen (**Table V**).

**Table IV.** Operating time (min)

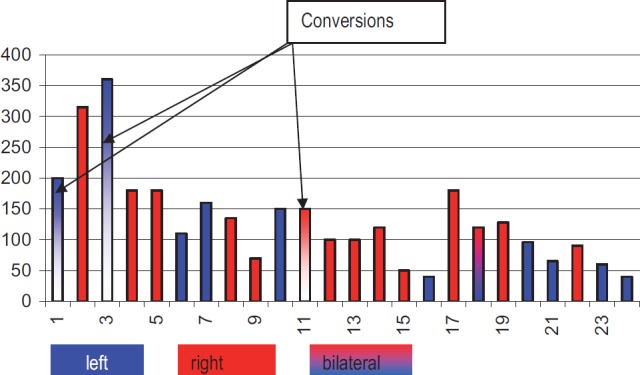

**Table V.** Conversion

	***Pathology***	***Specime n size cm***	***Side***	***Operating time (min)***	***Postop. stay (days)***
Adhesions	Cushing disease	3	Left	200	6
Spleen infarctisation	Conn syndrome	1,5	Left	360	7
Inflammatory adhesions and difficult mobilization	Cushing disease	5	Right	150	7

Blood lose was no more then 200 ml (30-200ml), so we did not use blood transfusions or substitute and all patients had a drainage for 24 hours. There were no complications, postoperative morbidity or mortality.

Demographic data for the patients who underwent conversion to open surgery are in **Table VI**.

**Table VI.** Demographic

	***Gender***	***Age***	***Previously surgery***	***Histology***
Adhesions	Women	35	Hiatal hernia	Adenoma
Spleen infarctisation	Women	55	Laparoscopic cholecystectomy	Hyperplasia
Inflammatory adhesions and difficult mobilization	Women	59	Laparoscopic cholecystectomy	Hyperplasia

From 23 patients and 24 procedures (1 patient with Cushing disease had 2 different procedures for left and right side) we had 25 adrenal glands removed (1 patient had a bilateral approach in the same operating time) with dimension between 1 and 6 cm as showed in **Table VII** and with a histology report found: 8 adenomas, 13 hyperplasia, 2 pheochromocytoma and 1 neoplasia.

**Table VII.** Specimen size (cm)

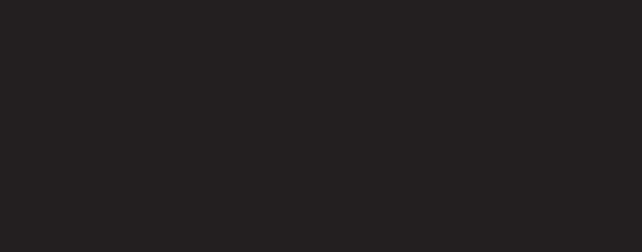

Postoperative hospital stay was no longer the same as for the patients with no previous abdominal surgery and pain medication was similar with every other laparoscopic procedure (except for the cases that needed conversion to open adrenalectomy). Median postoperative stay in our experience is of 4, 79 days (7) and for this study group of 4, 5 days (3-7 days) as in **Table VIII**.

**Table VIII.** Postoperative hospital stays (days)

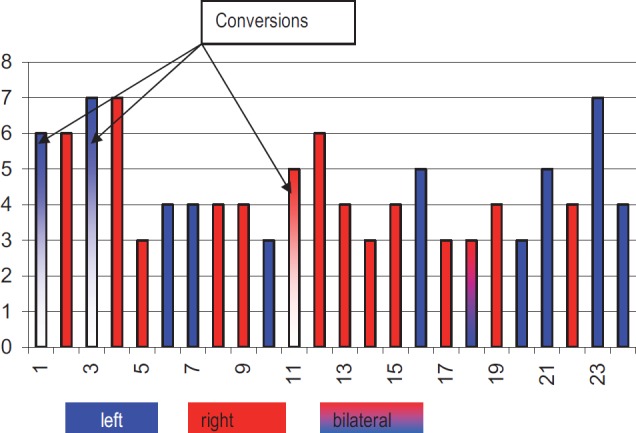

All our patients were clinically, biochemically evaluated post-surgery in “C. I. Parhon” National Institute of Endocrinology, and up to the present day, we have had no relapse or mortality in this study group.

## Discussion

Minimally invasive surgery changed adrenal surgery in past 2 decades although there is no consensus about the type of access either transperitoneal or retroperitoneal. In our clinic, the general surgeon’s team performed laparoscopic adrenalectomy and the only approach that we used for all our patients with adrenal pathology, except for the patients with general contraindication for laparoscopy, was anterolateral transperitoneal approach even for the patient with a previous abdominal surgery. Choosing the type of approach is related to the surgeon’s experience although 79-94% from the general surgeons prefer the transabdominal lateral approach [**8**] because there are clear and familiar anatomic landmarks and we can also explore the entire abdominal cavity in order to diagnose and treat other associated pathology.

Increasing the number of adrenalectomy from 9 in first year of our experience, although we are not an endocrine surgery department, up to 34 in 2008, is not a matter of increasing the adrenal pathology in our population, but an increase of diagnosis experience with highly performant imagistic methods and treatment of those diseases [**9**]. The reason we approached the laparoscopic way for almost all of our patients with adrenal pathology (with a very carefully preoperative preparation, especially for pheocromocytoma) is that we passed the learning curve and now, we can safely perform all the adrenal pathology and remove tumors which are larger than 10 cm.

In this study group, we did not significantly prolong the operating time because the adhesions and conversion rate were not higher than the ones for patients with no previous abdominal surgery, therefore, we did not believe that laparoscopic adrenalectomy performed by antero lateral transabdominal approach is contraindicated for patients with a previous abdominal surgery, even for open and major surgery in upper abdomen. The reasons for the conversion to open surgery were not different from the reasons for conversion to other pathologies laparoscopically approached and the most common are always the adhesions or the difficult mobilization.

The right moment for surgery is another problem to discuss. An accurate diagnosis and a proper preparation from the endocrinologist are essential for the success of the surgery, the same way as the postoperative follow-up. All the patients in this study were diagnosed in “C.I. Parhon” National Institute of Endocrinology after doing the CT scans, which were performed along with the biochemical measurements. The benefits of the minimally invasive surgery are important for these patients especially if we think that patients with Cushing disease or Cushing syndrome are more likely to become obese, and an open procedure can be complicated if we take into account the surgical procedure but also for the postoperative recovery and overall morbidity.

## Conclusions

Laparoscopic adrenalectomy is a challenging technical procedure that needs laparoscopic advanced skills to be performed safely. A better understanding of the anatomy, physiology and pathology of the adrenal along with a very careful pre and postoperative medication are essential for an outstanding outcome.

With an experienced surgical team, there is no difficulty in performing laparoscopic adrenalectomy by transperitoneal approach with no significantly prolonged operating time, even though the patients had a previous abdominal surgery.
